# Prevalence and associated factors of undernutrition among under-five children from model and non-model households in east Gojjam zone, Northwest Ethiopia: a comparative cross-sectional study

**DOI:** 10.1186/s40795-019-0290-y

**Published:** 2019-04-08

**Authors:** Abrehet Zeray, Getiye Dejenu Kibret, Cheru Tesema Leshargie

**Affiliations:** Department of Public Health, College of Health Science, Debre Markos University, PO box: 269, Debre Markos, Ethiopia

**Keywords:** Undernutrition, Under-five children, Model and non-model households, Ethiopia

## Abstract

**Background:**

Developing countries, undernutrition remains significant public health attention, as it was a combined consequence of poor dietary consumption and recurrent infectious illness especially in countries same Ethiopia. Undernutrition is associated with morbidity and mortality among children. This study, therefore, was conducted to assess the prevalence and associated factors of undernutrition among under-five children from the model and non-model households at Eastern Gojjam administrative Zone, northwest Ethiopia.

**Methods:**

A community-based comparative cross-sectional study was conducted from 1st July 2015 to 30th August 2015 in East Gojjam Zone among 507 households (170 from model-household and 337 from non-model household) selected using a multistage sampling technique. Data were collected using questionnaire and nutritional anthropometric measurement. The Emergency Nutrition Assessment for Standardized Monitoring and Assessment of Relief and Transition was used to convert raw anthropometric data into Z-scores. The collected data were entered into EpiData, and analysis was conducted using Statistical Package for Social Sciences (SPSS) version 22. The Emergency Nutrition Assessment for Standardized Monitoring and Assessment of Relief and Transition was used to convert raw anthropometric data into Z scores. Descriptive statistics were used to report the prevalence of outcome variable, undernutrition (intermesh of underweight, stunting, and wasting). In addition, results were presented using narration, tables, and figures including frequency and percentage. Adjusted Odds Ratio (AOR) with its 95% Confidence Interval (CI) was computed. Univariate and multivariate logistic regression analyses were done. A *p*-value less than 0.05 of was considered to declare a result as statistically significant.

**Results:**

This study found that the prevalence of undernutrition explained by stunting (height-for-age Z-score (HAZ) < − 2), underweight (weight-for-age Z-score (WAZ) < − 2) and wasting (weight-for-height Z-score (WHZ) < − 2) were 44.7% [95%CI 41.11, 48.29%]c, 15.3% [95%CI: 12.17, 18.43%] and 10% [95% CI 8.0, 12.0%], and 52.5% [95% CI: 48.62, 56.98%], 24.3% [95% CI: 20.22, 28.38%] and 11.3% [95% CI: 8.45, 14.15%] in under-five children among model household and non-model respectively. Protected water (AOR = 0.08, 95% CI: 0.03, 0.18) and less than three times daily intake of food (AOR = 4.06, 95% CI: 1.53, 10.82) were predictors for undernutrition among under-five model household. Protected source of drinking water (AOR = 0.07, 95% CI: 0.03, 0.13), households that ever-had education on complementary feeding (AOR = 0.19, 95% CI: 0.09, 0.25) and starting complementary feeding on 6 month after birth (AOR = 0.19, 95% CI: 0.09, 0.25) were significant predictors for under-five undernutrition among non-model households.

**Conclusion:**

The prevalence of undernutrition explained by stunting, underweight and wasting among under-five children in both model and non-model households were high. The prevalence of all the three parameters (stunting, underweight and wasting) was higher among the non-model households compared to the models, even if the differences were not statistically significant. Use of an unprotected source of drinking water and less than three times daily intake of food were found to be associated with undernutrition among under-five children in the model households. On the other hand, having educational exposure on complementary feeding, using drinking water from protected sources and initiation of complementary feedings at age of 6 months were found to be associated with undernutrition among children in the non-model households. Therefore, the concerned bodies must access safe and adequate water supply, works on information dissemination using mass media on timely initiation of complementary feeding, save water and on meal frequency should be strengthened.

**Electronic supplementary material:**

The online version of this article (10.1186/s40795-019-0290-y) contains supplementary material, which is available to authorized users.

## Background

Childhood undernutrition is widespread in low and middle-income countries. In these countries, it is an important and indirect cause of child mortality. Worldwide, stunting and wasting along with intrauterine growth restriction are responsible for nearly 2.1 million deaths in under five children that comprise 21% of all deaths [1]. Ethiopia has demonstrated promising progress in reducing levels of undernutrition over the past two decades. However, undernutrition is one of the major public health concerns among infants and young children in the country [2, 3]. The baseline levels of undernutrition remain high that the country still needs to continue substantial investment in nutrition [4]. In 2016, nearly 155 million children under the age of 5 years were suffering from stunting. Undernutrition contributes nearly 45% of deaths among children under the age of 5 years [5]. Globally a million children were underweight, 51 million were stunted and 17 million children were wasted in 2013 Moreover, undernutrition is attributed with inadequate feeding; faulty feeding practices, repeated infections like diarrheal diseases, acute respiratory infections and worm infestations [6–8]. Under-five children are the most vulnerable age group for undernutrition particularly the undernutrition in developing country like Ethiopia. The problem of undernutrition in the early stages of life can increase risk infections, morbidity, and mortality together with decreased mental and cognitive development [9]. Infectious diseases induced undernutrition; impacts of co-morbid diseases, diarrhea and pneumonia are factors revolving around child mortality. Having knowledge of problems distribution and determinants could contribute to design the management protocols of child health problems [10].

Previous studies have shown different factors associated with children undernutrition. These include: socio-demographic (mothers, education, birth interval, maternal age at childbirth, child sex, family size, maternal education status, and child age) [8, 11–15], biology (birth-weight), culture (feeding problems, meal frequency), economy (occupation of the mother), environment (poor hygienic and diseases (infections recurrent illness, measles, pertussis, and chronic disease) [14–16]. Additionally, low dietary intake, poor access to food, inequitable distribution of food within households, improper food storage and preparation, dietary taboos and infections with pathogens are also factors associated with the occurrence of undernutrition among under-five children [8, 11–13, 17].

Child undernutrition and mortality rates are sensible responses to both health programs and socioeconomic conditions such as income, unemployment, and illiteracy [18]. In Ethiopia, the prevalence of undernutrition is high [12, 14, 19, 20] which ranges from 14.6% in Addis Ababa to 46.3% in Amhara Region for stunting, 3.5% in Addis Ababa to 22.5% in Somalia region for wasting and 5% in Addis Ababa to 35.5% in Afar region for underweight [21]. The country started implementing health extension packages in which nutrition is one of the main components included in the health extension package. Based on the level of health extension package implementation, kebeles within the districts in the study area were categorized as a model and non-model households. Therefore, the objective of this study was to assess the prevalence of undernutrition and its associated factors among under-five children in the model and non-model Households of Gozamen district, northwest Ethiopia.

## Methods

### Study design, setting and study population

A community-based comparative cross-sectional study was conducted in East Gojjam Zone, northwest Ethiopia from 1st July to 30th August 2015. East Gojjam Zone is a suburban area in Amhara region, and found 300 km away from Addis Ababa, the capital city of Ethiopia. All women who have under-five children were included in this study.

### Sample size determination

The required sample size for the study was computed by Epinfo using two population proportion formula with the following assumptions: taking 80% power, 95% Confidence Interval (CI), and one to two ratio of model to non-model households. The proportion of undernutrition (46.9%) among model households was taken from a study conducted in rural parts of Tigray [12], prevalence two =66.9% (non-model) by taking if the difference was 20%, Where α = and the proportion of undernutrition among children from non-model households (66.9%) assuming if the difference was 20%. In addition, the design effect of 2 and 10% non-response rate was considered. Accordingly, a total of 507 (170 for Model households) and 337 for non-model households) were included in this study.

### Sampling technique and procedures

First, a two-stage stratified sampling (i.e., stratified by model and non-model households) used to select study participants. Then, from the existing 21 kebeles in the study settings, 6 of them were selected using lottery method. List of under-five children among from model and non-model household were extracted from community-based intervention for action (CBIA) data list in the selected kebeles. Second the calculated sample size was prevalence ally allocated to the model (n1 = 170) and non-model (n2 = 337) households. Third, the study participants were selected from the CBIA lists in each Kebele using simple random sampling method.

### Operational definitions

**Diarrheal disease:** is the passage of unusually loose or watery stools, usually at least three times in a 24 h period and the mother or caretaker said diarrhea.

**Model household:** A household graduated by the Ethiopian health extension program after fulfilling health extension packages and those who have graduation certificate.

**Wasting:** Weight-for-height measurement three Standard Deviation (SD) or more below the mean by taking world Health Organization (WHO) standard reference values which is called “wasted”.

**Stunting:** Children with height-for-age-z-score < − 2 SD as compared with the reference population were categorized as stunted.

**Underweight:** Children with weight-for-age-z-score < − 2 SD as compared with the reference population were categorized as underweight.

**Improved water supply:** It is a type of water source that, by nature of its construction or through active intervention, is likely to be protected from outside contamination, in particular from contamination with fecal matter [22].

**Improved toilet:** Is a facility that hygienically separates human excreta from human, animal and insect contact. Improved sanitation facilities include flush/pour-flush toilets or latrines connected to a sewer, septic tank or pit; ventilated improved pit latrines; pit latrines with a slab or platform of any material which covers the pit entirely, except for the drop hole; and composting toilets/latrines [22].

### Data collection technique and tools

Semi-structured English version questionnaire was developed from different related articles. The English version questionnaire was translated into the local language (Amharic) for data collection purposes and then back to keep the consistency. The anthropometric measurements were computed using data obtained by weight (balanced-beam scale and hanging spring scale) and height (measured using movable headboard) and age were taken from healthcare cards. For the weight measurement, all clothing including the diaper was removed and the infant was placed in the center of the calibrated weighing surface. Then reading the measurement to the nearest 0.5 g. For the height, we used a length-measuring device with a fixed headboard and a moveable footboard that are perpendicular to the surface on which the child is lying. A fixed measuring tape, marked in millimeters, was attached to the surface with the zero ends at the edge of the headboard. Then the first data collector was prepared the children by holding the head with crown against the headboard so that the child is looking straight upward. He also checks the trunk and pelvis are properly aligned with the measuring device while the second straighten the child’s legs and hold the ankles together with the toes pointed directly upward. And also move the footboard firmly against the soles of the feet. Finally, we recorded reading the measurement to the nearest 0.1 cm. In addition, edema data was collected by observation while other data were collected using an interview-administered questionnaire.

### Data collection procedure and quality

To assure the quality of data we performed a pretest. And also 1-day training was given for the data collectors and supervisors on the data collection producers, tools, and objectives of the study. Moreover, during the data collection activities, we routinely supervised the data collectors. Addition, the authors and an experienced data clerk entered and cleaned the data before the analysis.

### Data processing and analysis

The collected data were entered into EpiData version 3.1 and exported to SPSS version 22. Weight measuring scales were checked for accuracy and calibrated by using the known weight. Standard techniques were used during measuring the weight and height of the children. The Emergency Nutrition Assessment (ENA) for Standardized Monitoring and Assessment of Relief and Transition (SMART) was used to convert raw anthropometric data (weight, height, and age of children) into anthropometric Z-score (weight-for-age, height-for-age, and weight-for-height). WHO reference standard was used to classify children into levels of undernutrition (stunting, wasting and underweight). The result of Z-score was exported to SPSS analysis. Descriptive statistics were used to report the prevalence of outcome variable, undernutrition (intermesh of underweight, stunting, and wasting). Additionally, the findings were presented using narrative, tables, and figures including number and percent univariate and, multivariate logistic regression analysis implied. Variables with a *p*-value less than 0.2 during univariate logistic regression continued fitted to the multivariate logistic regression model. Adjusted Odds Ratio (AOR) with its corresponding 95% Confidence Interval (CI) was computed. Univariate *P*-value less than 0.05 were considered to declare a result as statistically significant.

## Results

### Socio-demographic characteristics

The necessary data were collected from a total of 507 (33.5% from model and 66.47% from non-model) households. All study participants were Orthodox Christian religion followers. The majority, 275 (81.6%) from non-model and 119 (70%) from model households were unable to read and write. Half of the participants, 85 (50%) and 250 (49.3%) from the model and non-model households were male by sex and nearly all participants, 490 (96.6%), 163 (95.9%) from model and 327 (97%) from non-model household were married (Table 1).

**Table 1:** Comparison of anthropometric results among model and non-model household under -five children in Gozamen district, Ethiopia, 2015

Nutritional status	Model households < 5 children	Non Model households < 5 children
Underweight	15.30% [95%CI: 12.17, 18.43%]	24.30%[95% CI: 20.22, 28.38%],
Stunted	44.70% [95%CI: 41.11, 48.29%],	52.50%[95% CI: 48.62, 56.98%],
Wasting	10.00% [95%CI: 8.00, 12.00%],	11.30%[95% CI: 8.45, 14.15%],

### Child caring practices

Slightly more than half of the participants, 102 (60%) from model and 183 (54.3%) from non-model households had antenatal care (ANC) service during the last pregnancy. Of the total study subjects, 74 (43.5%) from model households and 169 (50.1%) from non-model households use protected source of drinking water. In addition 72 (42.4%) in model households and 173 (51.3%) in non-model households had an education (Table 2).

**Table 2:** comparison of prevalence of underweight, stunting and wasting between Model households (*n* = 170) and non-model households (*n* = 337), with respect to socio-demographic of in Gozamen district, Ethiopia, 2015, Gozamen district, Ethiopia, 2015

Socio-demographic characteristics of participants		From model Households (*n* = 170)	From non-model Households (*n* = 337)	Prevalence (95%CI)
Sex of child	Male	85	165	49.31 [44.96, 53.67]
	Female	85	172	50.69 [46.33, 55.04]
Age of child in month	0–17	48	72	23.67 [20.16, 27.58]
	18–29	40	82	24.06 [20.53, 27.99]
	30–41	54	81	26.63 [22.95, 30.66]
	45–53	22	77	19.53 [16.29, 23.22]
	54–59	6	25	6.11 [4.33, 8.57]
Number of child	≤3	106	210	62.33 [58.01, 66.46]
	4–5	46	104	29.59 [25.76, 33.72]
	> 5	18	23	8.09 [6.0, 10.81]
Marital status	Married	18	23	96.65 [94.66, 97.91]
	Divorced	163	327	3.35 [2.09, 5.34]
Maternal education	Illiterate	119	275	43.39 [39.12, 47.76]
	Literate	51	62	56.61 [52.24, 60.88]
Monthly Income	≤600ETB	46	101	28.99 [25.20, 33.11]
	≤875ETB	34	73	21.11 [17.76, 24.89]
	≤1300ETB	46	84	25.64 [22.02, 29.64]
	> 300	44	79	24.26 [20.71, 28.20]

### Prevalence of stunting

**Fig. 1:**
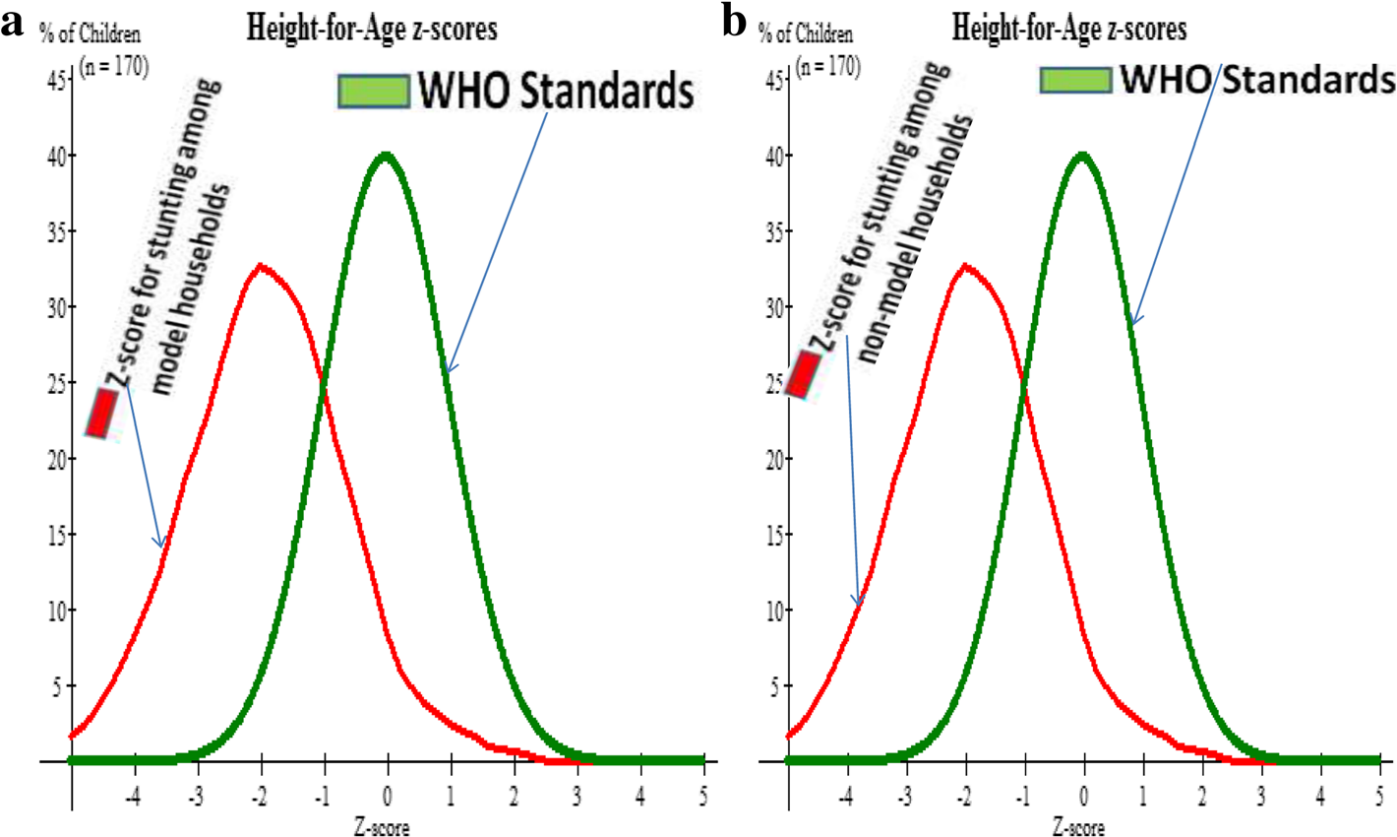
**a** and **b** Describes z-score curve together with WHO standard z-score curve for stunting (Height for age) of under-five children both in model households in Gozamen district, Ethiopia, 2015

In this study, 76 (44.7%) and 177 (52.7%) of under-five children among model and non-model households were stunted, respectively. The z-score curve including the WHO standard curve can be seen at (Fig. 1a and b).

### Prevalence of underweight

**Fig. 2:**
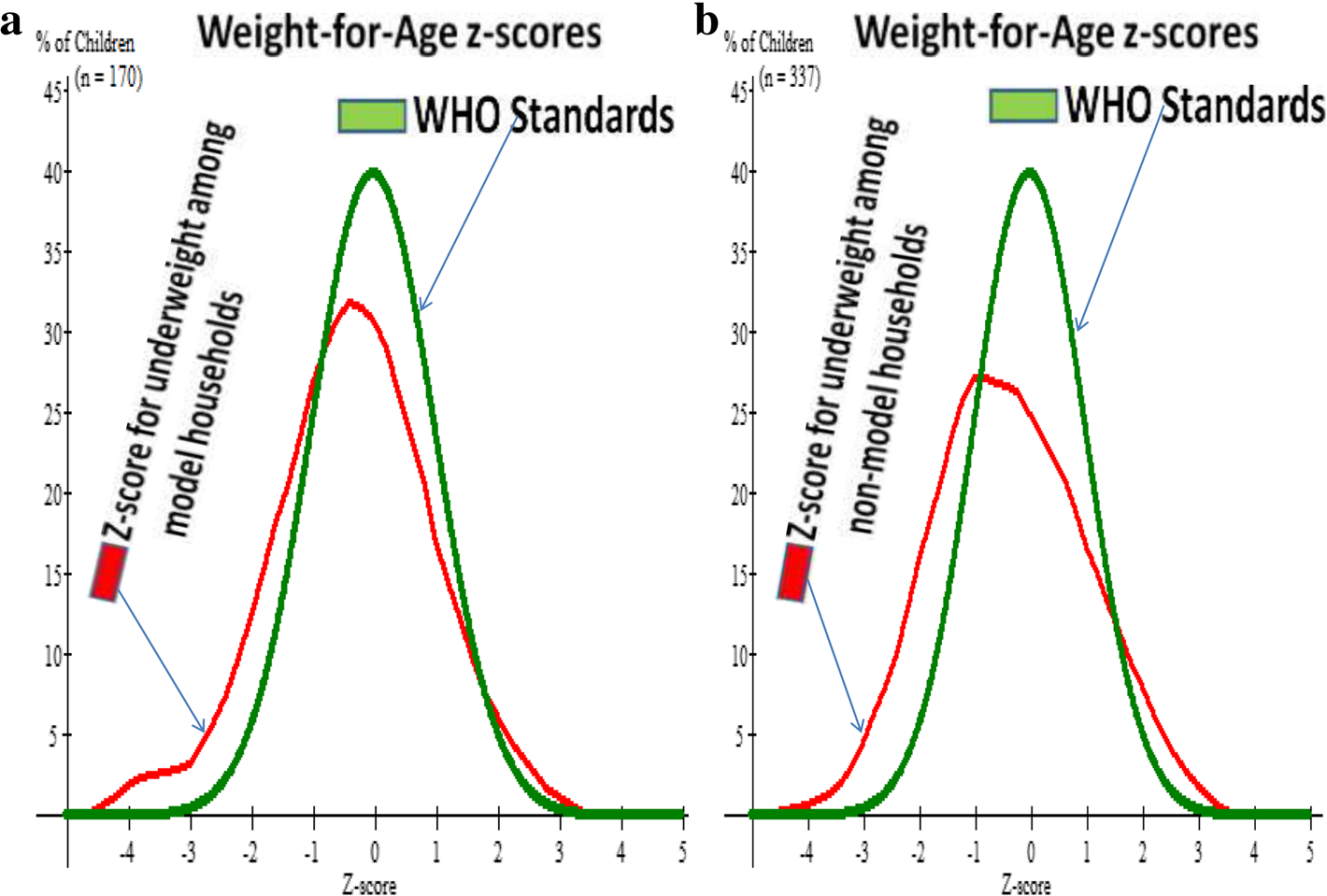
**a** and **b** Describes z-score curve together with WHO standard z-score curve for underweight (weight-for-age) of under-five children both in model households in Gozamen district, Ethiopia, 2015

Twenty six, (15.3%) and 82(24.3%) of under-five children in model and non-model households were underweight respectively. The z-score curve including the WHO standard curve can be seen at (Fig. 2a and b).

### Prevalence of wasting

In this study, 17(10%) and 38(11.3%) under five children in model and non-model households were wasted, respectively.

### Anthropometric results

By taking anthropometric results the overall prevalence of undernutrition was lower, 53.5% [95%CI: 49.32, 57.68%]) among under five children in model households as compared with 64.4% [95% CI: 59.51, 69.29%] in non-model households (Table 3).

**Table 3:** Distribution Environmental and other characteristics of respondents among model (*n* = 170) and non-model (*n* = 337) households in Gozamen district Ethiopia, 2015

Environmental and other characteristics		Types of households		Prevalence (95%CI)
		Model [N/%]	Non-model [N/%]	
ANC service	Yes	102 (60)	183 (54.3)	5621 [51.84, 60.49]
	No	68 (40)	154 (45.7)	43.79 [39.51, 48.16]
Source of drinking water	Protected water	74 (43.5)	169 (50.1)	47.93 [43.59, 52.30]
	Un protected	96 (56.5)	168 (49.9)	52.07 [47.70, 56.41]
Toilet	Present	159 (93.5)	211 (62.6)	73.00 [69.00, 76.90]
	Absent	11 (6.5)	126 (37.4)	27.00 [23.1, 31.00]
Hand wash facility	Present	132 (77.6)	228 (67.7)	71.01 [66.89,74.80]
	Absent	38 (22.4)	109 (32.3)	28.99 [25.20, 33.11]
Solid waste disposal Site	Present	151 (88.8)	119 (35.3)	56.20 [51.50, 60.70]
	Absent	19 (11.2)	218 (64.7)	43.80 [39.30, 48.50]
Exclusive Breast Feeding Education	Attended	94 (55.3)	126 (37.4)	43.39 [39.12, 47.76]
	Not attended	76 (44.7)	211 (62.6	56.61 [52.24, 60.87]
Complementary food education	Attended	72 (42.4)	173 (51.3)	48.32 [43.98, 52.69]
	Not attended	98 (57.6)	164 (48.7)	51.68 [46.92, 55.63]
Food distribution in house hold	Priority to child	81 (47.6)	166 (49.3)	48.72 [44.37, 53.08]
	Priority to other	89 (52.4)	171 (50.7)	51.28 [46.92, 55.63]
Two weeks Fever	Present	30 (17.6)	119 (35.3)	29.39 [25.57, 33.52]
	Absent	140 (82.4)	218 (64.7)	70.61 [66.48, 74.42]
Two weeks Diarrhea	Present	49 (28.8)	113 (33.5)	31.95 [28.02, 36.16]
	Absent	121 (71.2)	224 (66.5)	68.05 [63.84, 71.98]

### Factors associated with stunting

The univariate logistic regression in model households showed that a number of children, protected source of drinking water, Frequency of meal, starting complementary feeding and fever were associated with stunting. In non-model households’ sex of the households head, children used protected source of drinking water, complimentary food, education on complementary food, number of children and age of respondents were found to be associated with stunting. On the multivariate logistic regression, the use of a protected source of drinking water and frequency of meal taken by children were significantly associated with stunting among children in the model households. Children in the protected source of drinking water were less likely to be stunted as compared with the counterpart [AOR = 0.08; 95%CI: 0.03, 0.16]. In addition, children who get food less than three times per day were more than four times more likely to experience stunting compared with the counterpart, [AOR = 4.06; 95% CI: 1.53, 10.82]. The multivariate logistic regression analysis for children in the non-model households also showed that use of a protected source of drinking water and the timing of complementary feeding initiation was the factors associated with stunting. Children of families who are using a protected source of drinking water were less likely to be stunted compared with their counterparts [AOR = 0.07; 95%CI: 0.03, 0.13]. Children who started complementary feeding on 6 months were less likely to be stunted than those who started other than 6 months [AOR = 0.19; 95% C.I:0.09, 0.25]. Children whose mother had education on complementary food were less likely to be stunted than their counterparts [AOR = 0.19; 95% C.I:0.09, 0.25] (Additional file 1).

### Factors associated with underweight

The univariate logistic regression analysis in model households showed food distribution, daily meal frequency, the source of drinking water and family income were associated with underweight. In non-model households, women power using money, ANC service utilization, protected source of drinking water, food distribution, child liquid waste disposal, and solid waste disposal were associated with underweight.

Multivariate logistic regression analysis for children in model households showed that the source of drinking water and daily meal frequency were significantly associated to be underweight. Children of household using a protected source of drinking water were less likely to be underweight compared to their counterparts [AOR = 0.19; 95% CI: 0.07, 0.25]. Children daily intake of food less than 3 meals per day were 3 times more likely to be underweight compared to children daily intake 3 and above meals per day [AOR = 3.11;95% CI:1.11, 8.73]. In non-model household under five children mothers attending antenatal care service during pregnancy were less likely to be underweight compared to the counterpart [AOR = 0.39;95%CI: 0.23, 0.66]. Children who had access to a protected source of drinking water were less likely to be underweight compared to the counterpart [AOR = 0.59; 95% CI: 0.35, 0.99]. During food distribution, non-model household given priority to their children were less likely to be underweight [AOR = 0.39; 95%CI: 0.21, 0.73] compared to the counterpart (Additional file 2).

### Factors associated with wasting

During univariate logistic analyses, ANC service, source of drinking water, complementary feeding at 6 months, priority to feed their children and complimentary food education were significantly associated with wasting in the model household. In this household, children who start complementary feeding at 6 months were less likely to be wasted compared 6 months and above [AOR = 0.27; 95% CI: 0.08, 0.89]. Model household those who give priority to feeding their children were less likely to be wasted compared to the counterpart [AOR = 0.20; 95%CI: 0.06, 0.67].

Similarly, in non-model households, the Univariate logistic regression, complimentary food start, breastfeeding education, ANC service, solid waste disposal, and household incomes were significantly associated with wasting. In the multivariate analysis, a household with ANC service and solid waste disposal were significantly associated with wasting. Children from mothers who had ANC service were less likely to be wasted compared to the counterpart [AOR = 0.22; 95% CI: 0.09, 0.53]. Under five children who from household those who have solid waste disposal site were less likely to be wasted compared to the counterpart [AOR = 0.42; 95% CI: 0.21, 0.85] (Additional file 3).

## Discussion

Undernutrition is the most critical problem in the developing country. In Ethiopia, especially in the Amhara region, half of the under-five children are suffering from undernutrition [2, 20]. This study aimed to determine the prevalence of undernutrition and associated factors in East Gojjam Zone, northwest Ethiopia.

This study found that the prevalence of undernutrition among under-five children was high. Particularly, the percentage of stunting was (44.7%), underweight (15.3%) and wasting (10%) among model households. For children of non-model households, the prevalence of stunting was 52.5%, underweight (24.3%) and wasting (11.3%). In addition, significant variation was observed in the percentage of undernutrition among children between model and non-model households. The prevalence of undernutrition was higher among children in the non-model compared to children in the model families. This might be due to the difference in economic and agricultural imputes. This finding is supported by previous studies conducted in Bule Hora, Ethiopia [6], Hidabu Abote district [23], Haramaya district of Ethiopia [24], the Ethiopian Demographic Health Survey [2], in Wollo, Amhara region [14] and Tigray region [19]. Moreover, these might be due to that the study settings are similar in socio-demographic characteristics, residence and socio-economic status.

This study also showed a higher percentage of undernutrition compared with previous studies conducted in Gumbirt, northwest Ethiopia [20], in Egypt and Kenya [25]. This might be due to the difference in the sample size and study period between the current and previous studies. Moreover, the difference observed in undernutrition might be due to the difference in the socio-economic, feeding, lifestyle and socio-demographic characteristics. Similarly, Egypt [20] and Kenya [25] are better in economic development as compared with Ethiopia. The prevalence of underweight and wasting reported in this study was lower compared with the study done in Gumbirt, northwest Ethiopia [20] and Kenya [25]. This might be due to the difference in the sample size, study setting, and socio-demographic characteristics. In contrary, the current study comes with lower prevalence s of underweight as compared with a study conducted in Wollo, Amhara region [14], Tigray region [12], Hidabu Abote district [23] and Haramaya district [24] of Ethiopia. The discrepancy might be due to the difference in the study period and sample size. Similarly, lower prevalence of wasting also reported by the current study as compared with the previous study reported in Tigray, Hidabu Abote district and Haramaya district of Ethiopia [12, 23, 24]. Moreover, stunting was higher compared with the previous finding reported by Kenya [26].

This study also focused on identifying factors that have the association with undernutrition status of under-five children both among model and non-model households. In the binary variable of model households, the number of children, protected source of drinking water, daily meal frequency, complimentary food start, and fever were associated with stunting. Whereas in non-model households’ sex, protected source of drinking water, complimentary food, education on complementary food, number of children and age of caregiver were found to be associated with stunting. Moreover, the binary variable of model households, food distribution; daily meal frequency, the source of drinking water and family income were associated with underweight. Whereas, in non-model households, the power to use the money, ANC service utilization, protected source of drinking water, food distribution, child waste disposal, and solid waste disposal were associated with underweight in non-model households. In addition, the binary variable of model households, ANC service, source of drinking water, complementary feeding at 6 months, priority to feed their children and complimentary food education were significantly associated with wasting in the model household. Whereas, in non-model households, complimentary food start, breastfeeding education, ANC service, solid waste disposal, and household incomes were significantly associated with wasting.

Non-protected source of water identified as a predictor for the occurrence of under-five children under-nutrition/stunting/ among both model and non-model households. This might be that water from a contaminated source serves as a source for different parasitic and gastrointestinal infection. These infections expose children for undernutrition (stunting). According to the WHO, at least 50% of the combined undernutrition in children is related to unsafe water, inadequate sanitation or insufficient hygiene [22]. This can be supported by previous evidence observed by a study done in Medabazana district North Ethiopia [12].

Moreover, in this study daily meal frequency was also identified as a significant factor for the occurrence of undernutrition. Children who took meal frequency less than three times per day were more likely to be undernourished as compared to the counterparts. This can supported by study finding reported in Egypt [25].

Similarly, a lack of education on complementary feeding practice contributes 61% for the occurrence of under-five children under-nutrition/stunting/ among non-model households compared with their counterparts. This might be due to a mother who knows the recommended complementary feeding practice might have a better commitment to start complementary feeding on the recommended time and continue [1]. This supported by previous research conducted in the Gumbirt district northwest Ethiopia [14].

Children who start complementary feeding at 6 months were less likely to be wasted as compared with the counterpart. This finding is comparable with study finding reported in Bule Hora district south Ethiopia [15]. This similarity might be due to the similarity in socio-demographic characteristics particularly on community awareness towards timely initiation of complementary feeding. Information on complementary feeding is crucial for starting complementary food based on the recommended date [1].

Furthermore, the source of drinking water was also identified as significant predictors for an increased odd of undernutrition (stunted and underweight) among under-five children who were from both model and non-model household. Under-five children from model and non-model households, who use non-protected water, were 92 and 93% times more likely to be undernutrition as compared with their counterparts. This finding is comparable with previous study report done by Machakel district.

The timing of complementary feeding was also recognized as predictors for the high prevalence of undernutrition among model households. Children who came from model households and those who did not start complementary feeding on 6 months had a 73% (AOR = 0.27, 95%CI: 0.08, 0.89) higher odds of undernutrition/wasting/ as compared to their counterparts. Children who came from model households and those who served prior than elders had 80% (AOR = 0.20, 95%CI: 0.06, 0.67) lower odds of undernutrition as compared to their counterparts.

Antenatal care service and solid waste disposal system were also identified as factors for the increased odds of undernutrition among children in non-model households. Non-model households, who had no ANC follow up had 80% (AOR = 0.20, 95%CI: 0.06, 0.67) higher odds of undernutrition compared to their counterparts. Similarly, children from non-model households and those who had no solid waste disposal were 58% (AOR = 0.420, 95%CI: 0.19, 0.91) times more likely to be undernutrition compared with the counterpart. This fact supported well by research findings reported by Nigeria [27]. This might be due to those women who have antenatal follow-up provided education on focusing on the recommended infant feeding practice. As a result, she can feed her child based on the information gained from health facilities. Moreover, most of the time women who have ANC follow-up are those with higher education and economic status.

## Limitation of the study

This study recognized the following limitations: There may be a recall bias during data collection time that might be increased or decrease the prevalence of undernutrition. Moreover, this study did not address some factors as per UNICEF framework. As a result of disaggregate analyses about associated factors the wider confidence intervals were experienced.

## Conclusions

This study showed a higher prevalence s of undernutrition that is explained by stunting, wasting and underweight among under-five children who were from non-model households compared to children from model households. Moreover, the prevalence of undernutrition was higher compared with previous national pieces of evidence and studies finding reported in the country. Among model household, this high prevalence of undernutrition was associated with a drinking water source (not- protected) and daily food intake frequency (less than three times). Similarly, among non-model household, this high prevalence of undernutrition was associated with a drinking water source (not- protected) and education (those who had no education on complementary food and starting complementary food on 6 months). Therefore as a recommendation, the concerned bodies must access safe and adequate water supply, works on information dissemination using mass media about complementary feeding, save water and on meal frequency. The community should be encouraged to use drinking water from protected sources. Awareness creation on complementary feeding practice and timely initiation at 6 months should be strengthening in the community.

## Additional files

Additional file 1: Bivariate and Multivariate analysis of factors associated with stunting for model and non-model households under-five children in Gozamen district, Ethiopia, 2015. (DOCX 18 kb)

Additional file 2: Bivariate and Multivariate analysis of factors associated with underweight for model and non-model households under-five children in Gozamen district, Ethiopia, 2015. (DOCX 18 kb)

Additional file 3: Bivariate and Multivariate analysis of factors associated with wasting for model and non-model households under-five children in Gozamen district, Ethiopia, 2015. (DOCX 14 kb)

## Abbreviations

ANC: Antenatal Care
AOR: Adjusted Odds Ratio
BMI: Body Mass Index
CBIA: Community-Based Intervention for Action
ENA: Emergency Nutrition Assessment
SD: Standard Deviation
SMART: Standardized Monitoring and Assessment of Relief and Transition
WHO: World Health Organization
