# Supplementary material for: Prevalence and associated factors of undernutrition among under-five children from model and non-model households in east Gojjam zone, Northwest Ethiopia: a comparative cross-sectional study
Source: BMC Nutr. 2019 Apr 8;5:27. doi: 10.1186/s40795-019-0290-y (PMC7050904; doi:10.1186/s40795-019-0290-y)
Supplement: Supplementary file 1 — Bivariate and Multivariate analysis of factors associated with stunting for model and non-model households under-five children in Gozamen district, Ethiopia, 2015. (DOCX 18 kb) [file 40795_2019_290_MOESM1_ESM.docx]

| **Variables** | **Stunting among model Households** | | | | **Stunting among non-model Households** | | | |
| --- | --- | --- | --- | --- | --- | --- | --- | --- |
|  | **No** | **yes** | **COR (95% CI)** | **AOR (95% CI)** | **No** | **Yes** | **COR (95% CI)** | **AOR (95% CI)** |
| **Source of drinking water** | N (%) | N (%) |  |  | N (%) | N (%) |  |  |
| Protected | 20(21.3) | 54(71.1) | 0.11(0.055,0.22) | 0.08(0.03, 0.18) | 38(23.7) | 131(74) | 0.11(0.07,0.18) | 0.07(0.03,0.13) |
| Unprotected | 74(78.7) | 22(28.9) | 1.00 | 1.00 | 122(76.3) | 46( 26) | 1.00 | 1.00 |
| **Complementary food start** | | | | |  |  |  |  |
| Started at 6 month | -------------- | ------------- | -------------- | -------------- | 51(31.9) | 122(68.9) | 0.21(0.13,0.33) | 0.19(0.09,0.25) |
| Started before and after six month | -------------- | ------------- | -------------- | -------------- | 109(68.1) | 55(31.1) | 1.00 | 1.00 |
| **Frequency of food intake per day** | | | |  |  |  |  |  |
| < 3 meal per day | 70(92.11) | 67(71.3 ) | 4.70(1.826,12,107) | 4.06(1.53,10.82) | -------------- | ------------ | -------------------- | ------------------ |
| ≥ 3 meal per day | 6(7.89) | 27(28.7) | 1.00 | 1.00 | -------------- | ------------ | -------------------- | ------------------ |
| **Complementary food education** | | |  |  |  |  |  |  |
| **Attended** | -------------- | ------------ | -------------------- | ------------------ | 55(33.54) | 122(70.5) | 0.21(0.10,0.27) | 0.19(0.09,0.25) |
| **Not attended** | -------------- | ------------ | -------------------- | ------------------ | 109(66.46) | 51(29.5) | 1.00 | 1.00 |
