# Supplementary material for: Prevalence and associated factors of undernutrition among under-five children from model and non-model households in east Gojjam zone, Northwest Ethiopia: a comparative cross-sectional study
Source: BMC Nutr. 2019 Apr 8;5:27. doi: 10.1186/s40795-019-0290-y (PMC7050904; doi:10.1186/s40795-019-0290-y)
Supplement: Supplementary file 2 — Bivariate and Multivariate analysis of factors associated with underweight for model and non-model households under-five children in Gozamen district, Ethiopia, 2015. (DOCX 18 kb) [file 40795_2019_290_MOESM2_ESM.docx]

| **Variables** | **Underweight among model Households** | | | | | **Underweight among non-model Households** | | | | |
| --- | --- | --- | --- | --- | --- | --- | --- | --- | --- | --- |
|  | **No** | **Yes** | **COR (95% CI)** | **AOR (95% CI)** | **P-value** | **No** | **Yes** | **COR (95% CI)** | **AOR (95% CI)** | **P-value** |
| **Source of Drinking Water** | | | |  |  |  |  |  |  |  |
| Protected | 55(38.2) | 19(73.1) | 0.23(0.90,0.58) | 0.19(0.07,0.25) | 0.01 | 127(49.8) | 42(51.2) | 0.94(0.57,2.01) | 0.59(0.35,0.99) | 0.048 |
| Unprotected | 89(61.8) | 7(26.9) | 1 | 1 |  | 128(50.2) | 40(48.8) | 1 | 1 |  |
| **Frequency of food intake** | | | |  |  |  |  |  |  |  |
| < 3 meal per day | 119(82.6) | 18(69.2) | 2.12(0.83,5.40) | 3.11(1.11,8.73) | 0.032 | ----------- | ------------- | ----------------- | ------------------ |  |
| ≥ 3 meal per day | 25(17.4) | 8(30.8) | 1.00 | 1.00 |  | --------- | --------- | ------------------- | ------------------ |  |
| **Food distribution** |  |  |  |  |  |  |  |  |  |  |
| Priority to child | ----------- | ----------- | ----------------- | ------------------ |  | 118(46.3) | 48(58.5) | 0.61(0.37,0.91) | 0.39(0.21,0.73) | 0.043 |
| Priority to other | --------- | --------- | ------------------- | ------------------ |  | 137(53.7) | 34(41.6) | 1.00 | 1.00 |  |
| **ANC service** |  |  |  |  |  |  |  |  |  |  |
| Yes | ----------- | ----------- | ----------------- | ------------------ |  | 127(49.8) | 56(68.3) | 0.46(0.27,0.78) | 0.39(0.29,0.66) | 0.001 |
| No | --------- | --------- | ------------------- | ------------------ |  | 128(50.2) | 26(31.7) | 1.00 | 1.00 |  |
