# Supplementary material for: Prevalence and associated factors of undernutrition among under-five children from model and non-model households in east Gojjam zone, Northwest Ethiopia: a comparative cross-sectional study
Source: BMC Nutr. 2019 Apr 8;5:27. doi: 10.1186/s40795-019-0290-y (PMC7050904; doi:10.1186/s40795-019-0290-y)
Supplement: Supplementary file 3 — Bivariate and Multivariate analysis of factors associated with wasting for model and non-model households under-five children in Gozamen district, Ethiopia, 2015. (DOCX 14 kb) [file 40795_2019_290_MOESM3_ESM.docx]

| Variables | Wasted among model Households | | | | Wasted among non-model Households | | | |
| --- | --- | --- | --- | --- | --- | --- | --- | --- |
|  | **Yes (%)** | **No (%)** | **COR (95% CI)** | **AOR (95% CI)** | **Yes (%)** | **No (%)** | **COR (95% CI)** | **AOR (95% CI)** |
| Complementary food start | |  |  |  |  |  |  |  |
| Equal to six month | 13(76.5) | 80(52.89) | 0.34(0.11,1.08) | 0.27(0.08,0.89) | -------- | ------ | ------------------ | ------------------- |
| < or > six month | 4(23.5) | 73(47.71) | 1.00 | 1.00 | -------- | ------ | ------------------ | ------------------- |
| Food distribution |  |  |  |  |  |  |  |  |
| Priority to child | 13(76.5) | 68(44.4) | 0.25(0.77,0.79) | 0.20(0.06,0.67) | --------- | ------- | ------------------- | -------------------- |
| Priority to other | 4(23.5) | 85(55.6) | 1.00 | 1.00 | --------- | ------- | ------------------- | -------------------- |
| Solid waste disposal |  |  |  |  |  |  |  |  |
| Yes | --------- | ------- | ------------------- | ------------------- | 22(58.9) | 112(37.5) | 0.44,(0.22,0.86) | 0.42,(0.19,0.91) |
| No | --------- | ------- | ------------------- | ------------------- | 16(42.1) | 187(62.5) | 1.00 | 1.00 |
| ANC service |  |  |  |  |  |  |  |  |
| Yes | --------- | ------- | ------------------- | ------------------- | 13(76.5) | 68(44.4) | 0.25(0.77,0.79) | 0.20(0.06,0.67) |
| No | --------- | ------- | ------------------- | ------------------- | 4(23.5) | 85(55.6) | 1.00 | 1.00 |
